# Control beliefs can predict the ability to up-regulate sensorimotor rhythm during neurofeedback training

**DOI:** 10.3389/fnhum.2013.00478

**Published:** 2013-08-15

**Authors:** Matthias Witte, Silvia Erika Kober, Manuel Ninaus, Christa Neuper, Guilherme Wood

**Affiliations:** ^1^Department of Psychology, University of Graz,Graz, Austria; ^2^Laboratory of Brain-Computer Interfaces, Institute for Knowledge Discovery, Graz University of TechnologyGraz, Austria

**Keywords:** EEG, locus of control, neurofeedback, performance prediction, sensorimotor rhythm

## Abstract

Technological progress in computer science and neuroimaging has resulted in many approaches that aim to detect brain states and translate them to an external output. Studies from the field of brain-computer interfaces (BCI) and neurofeedback (NF) have validated the coupling between brain signals and computer devices; however a cognitive model of the processes involved remains elusive. Psychological parameters usually play a moderate role in predicting the performance of BCI and NF users. The concept of a locus of control, i.e., whether one’s own action is determined by internal or external causes, may help to unravel inter-individual performance capacities. Here, we present data from 20 healthy participants who performed a feedback task based on EEG recordings of the sensorimotor rhythm (SMR). One group of 10 participants underwent 10 training sessions where the amplitude of the SMR was coupled to a vertical feedback bar. The other group of ten participants participated in the same task but relied on sham feedback. Our analysis revealed that a locus of control score focusing on control beliefs with regard to technology negatively correlated with the power of SMR. These preliminary results suggest that participants whose confidence in control over technical devices is high might consume additional cognitive resources. This higher effort in turn may interfere with brain states of relaxation as reflected in the SMR. As a consequence, one way to improve control over brain signals in NF paradigms may be to explicitly instruct users not to force mastery but instead to aim at a state of effortless relaxation.

## Introduction

In the last twenty years of neuroimaging research a clear view has emerged that patterns of brain activity can be directly linked to different cognitive states. Users of neurofeedback (NF) can learn to modulate their brain signals over several training sessions. Alternatively, using multivariate analysis methods one can try to decode these brain states and use the output of a classifier to control external devices in a so-called brain-computer interface (BCI). A key concept behind all these approaches is the assumption that the brain activations associated with a cognitive state are stable over time, highly specific and distinct from other states. However, these prerequisites are not always fulfilled and the neural correlates of a cognitive state are masked by various sources of physiological and environmental noise. The search for reliable predictors of performance therefore remains one of the major challenges in this field of research.

To achieve a reliable readout of brain patterns most BCI studies have put the primary load on the machine-learning side. This seems a straightforward approach as the goal of many BCIs is to detect overt brain states, like decoding movement parameters or stimulus-evoked activity (for review see: Donoghue, 2008; Green and Kalaska, 2011). Here, a standard procedure is to adapt the classification across participants and sessions (McFarland et al., 2005; Shenoy et al., 2006; Blumberg et al., 2007; Vidaurre et al., 2007). In contrast, NF is inspired by conditioning and often modulates a covert, unconscious state by immediate reward. One of the best described examples is the voluntary regulation of slow cortical potentials in healthy participants and paralyzed patients (Birbaumer et al., 1999; Kubler et al., 1999, 2001; Neumann and Birbaumer, 2003). It has been shown that self-regulation of these brain signals is optimally learned without giving definite strategies (Birbaumer et al., 1988; Neumann et al., 2003). An issue in this design is that users may feel lost at early stages of training and start to explore different ways to regulate their brain activity. Due to the immediate closed-loop feedback this “trial-and-error” learning can result in progressively better control and is believed to ultimately lead to an automated skill (Wolpaw and McFarland, 1994; Neumann and Birbaumer, 2003; Hinterberger et al., 2005). However, the literature has also described a significant proportion of people who are unable to gain control over signals in BCI and NF paradigms (Guger et al., 2003; Kübler et al., 2004; Nijboer et al., 2008; Blankertz et al., 2010b). The reasons for this phenomenon of “illiteracy” are still unknown and only few studies tried to assess predictors of successful performance. Factors like mood, motivation, intelligence and personal traits have been reported to show only moderate correlations to performance in healthy and impaired participants (Nijboer et al., 2008, 2010; Kleih et al., 2010; Hammer et al., 2012). Yet there is evidence from neuropsychological tests that part of the variations seen in NF training may be connected to memory and attentional abilities of participants (Roberts et al., 1989; Daum et al., 1993; Holzapfel et al., 1998). In particular, fronto-parietal gamma-band activity has been reported to influence sensorimotor activity, presumably reflecting attentional networks (Grosse-Wentrup and Schölkopf, 2012). Furthermore, the initial performance level was shown to have some predictive value for future performance (Neumann and Birbaumer, 2003; Kübler et al., 2004). These results may thus suggest that the overall, not necessarily task-related, cognitive resources impact the level of control in NF and BCI tasks.

Given that all experiments in the field of BCI and NF imply the use and interaction with technologic environments it is surprising that only one study directly assessed how technology commitment may impact performance (Burde and Blankertz, 2006). The authors evaluated the “locus of control of reinforcement” (LOC), a psychological construct developed by Rotter’s social learning theory (Rotter, 1966). According to this theory, people with an external LOC tend to attribute the result of their own actions to external sources like luck, chance or unpredictable circumstances. Conversely, an internal LOC describes the personal trait to link results and own actions and thus people feel that they are in control of the situation. Burde and Blankertz (2006) assessed the general control belief with an IPC (Internal, Powerful Others and Chance) questionnaire (Krampen, 1981) and the specific interaction with technology as indexed by the KUT (Kontrollueberzeugug im Umgang mit Technik), i.e., control beliefs while dealing with technology (Beier, 1999, 2004). They reported a positive correlation between KUT scores and BCI performance in 12 healthy participants partaking in a motor imagery task. However, only a single session was recorded and the features for classification were individually adapted for each participant.

We therefore sought to clarify whether control beliefs while dealing with technology, as reflected in the KUT score, can predict performance in NF training over several sessions. To this end, 10 participants trained to gain control over their sensorimotor rhythm (SMR, 12–15 Hz) in 10 sessions spanning up to five weeks via real-time visual feedback. An additional control group of 10 participants took part in the same protocol but received sham visual feedback. SMR is known to originate from thalamo-cortical loops and increased SMR amplitude is found during states of relaxed wakefulness with reduced sensory and motor excitability (Gruzelier et al., 2006; Serruya and Kahana, 2008). Because SMR desynchronizes during movement execution and during motor imagery in an event-related manner (Pfurtscheller and Neuper, 1997; Pfurtscheller and Lopes Da Silva, 1999; McFarland et al., 2000), it has been extensively used in BCI research (Cincotti et al., 2003; Pfurtscheller et al., 2006; Mellinger et al., 2007; Blankertz et al., 2010a). The opposing effect of voluntarily increasing SMR amplitude can also be learned through NF training (Vernon et al., 2003; Egner et al., 2004; Hoedlmoser et al., 2008; Weber et al., 2011; de Zambotti et al., 2012). However, only few studies investigated the changes of SMR over longer time periods of training and, to our knowledge, there is no report on the influence of technology commitment in training scenarios.

## Materials and Methods

### Participants

Twenty healthy participants (10 males, mean ± SD age: 24.4 ± 1.8 years) participated in this study after giving written informed consent. The study was approved by the local ethical committee of the University of Graz in accordance to the Declaration of Helsinki. No participant had any experience in NF- and BCI-paradigms prior to this study. In a double-blinded approach participants were randomly assigned to one group of 10 participants: either receiving real visual feedback coupled to brain rhythms experimental group (EG) or receiving a video of sham feedback randomly taken from one of these real feedback sessions control group (CG).

### Experimental paradigm

We recorded brain signals with a 16 Ag/AgCl electrode system (g.USBamp, g.tec medical engineering GmbH, Austria) mounted according to the International 10–20 EEG system and referenced to the left mastoid. Ground electrode was set on Fpz electrode and signals digitized at a sampling frequency of 256 Hz. In addition, electrooculography (EOG) was recorded to eliminate eye movement artifacts post-hoc.

Online visual NF was implemented via SIMULINK software (The MathWorks, Natick, USA). Raw signals were band-pass filtered in the respective target bands (precise frequencies see below; 6th order butterworth IIR) and squared to obtain power estimates. To ensure a smooth visual feedback we then applied a moving average of 256 samples and updated the computer screen at a rate of 10 Hz. The feedback design was adopted from a previous study (Weber et al., 2011): while a central bar was coupled to the user’s absolute power of the SMR (12–15 Hz) recorded at electrode Cz, two smaller flanking bars reflected absolute power in *θ* (4–7 Hz) and *β* (21–35 Hz) ranges at Cz, respectively. This setup of three moving bars was chosen to ensure voluntary regulation of SMR and at the same time minimize influence of eye movements (*θ*), muscle activations and other task-unrelated components (*β*). Each of the 10 sessions started with a first baseline run (3 min) where participants were instructed to relax and watch the moving feedback bars coupled to their brain activity without trying to control them. Then six feedback runs (3 min each) were recorded and participants tried to gain voluntary control over their brain rhythms, i.e., an increase of power was associated with an increase of the feedback bar.

Participants’ task was to increase the height of the central bar and at the same time keep the two smaller bars as low as possible. To facilitate the recognition of current performance, participants received an additional rewarding feedback whenever the bigger central bar reached a pre-defined threshold without concomitant artifacts: a number in the middle of the bar served as reward counter and was incremented by one unit each time this target state was achieved for 250 ms (i.e., between 0 and 720 points could be earned per run). The individual threshold was initially determined on the median absolute SMR power of the baseline run and progressively adapted using the median power of each previous feedback run. Similarly, the small flanker bars were calibrated once on the baseline recording of each day (threshold: mean power + 1 SD) and feedback bars changed color from green to red whenever influence of artifacts reached the individual thresholds.

### Data analysis

All preprocessing and data analysis of EEG recordings were performed offline using the Brain Vision Analyzer software (version 2.01, Brain Products GmbH, Munich, Germany). First, 1 s epochs of data were inspected for eye movement artifacts by a trained investigator and contaminated epochs were manually rejected. Next, we applied an automated rejection of additional artifacts like muscular activity, high-frequency noise or drifts (rejection criteria: step >50.00 μV per sampling point, absolute voltage value >120.00 μV).

In line with past studies (Weber et al., 2011; de Zambotti et al., 2012), absolute values of SMR power in a fixed range (12–15 Hz) were calculated for all epochs of length 1 s using Brain Vision Analyzer’s built-in method of complex demodulation. For each run, this procedure outputs mean SMR power over the whole time window of 3 min.

### Locus of control of reinforcement

The LOC was assessed in the context of dealing with technology by the KUT questionnaire (Beier, 1999, 2004). This one dimensional construct of LOC is a subjective 5-point Likert scale rating that considers actual technologic biography in eight items (range of total score: 8–40). The questionnaire is available in German and has a high reliability. To monitor control beliefs over time each participant was asked twice, before the first and after the 10^th^ training session.

### Statistical data analysis

Absolute SMR power values were calculated for each run separately and mean power was tested for differences using a 2 × 3 × 2 repeated measures ANOVA with between-factor group (EG vs. CG) and within-factors session (sessions 1–3, sessions 4–7, sessions 8–10) and run (runs 2–4, runs 5–7). Measures of effect size were reflected in partial eta-squared (*η*^2^) and observed power (*Obs_pow_*). Post-hoc tests, if necessary, were run on significant effects using Fisher’s Least Significant Difference (LSD) test.

To report trends of power changes we used a linear fit and assessed the significance of the regression slope using t-statistics of the regression model implemented in MATLAB (The MathWorks, Natick, USA). Group-wise comparisons of power and KUT values were assessed using paired t-test. All statistics considered a nominal probability level of *p* < 0.05 significant.

## Results

### Control beliefs and neurofeedback training

As a first step, we quantified the distribution and changes of our predictor variable across the population of participants. Our assessment of control beliefs while dealing with technology before NF training revealed no differences (*t*(18) = −0.15, *p* = 0.88 n.s., paired t-test) in KUT scores between the EG using real NF and the CG that relied on sham feedback. Overall scores were rather high varying only little around a value of 33 on average. Next, we intended to characterize differences within the groups in more detail. When dividing each group into subgroups using median-split, significant differences of 7.42 and 7.2 scores on average were observed between subgroups of high and low KUT within EG and CG respectively (*t*(8) = −4.82 for the EG and *t*(8) = −4.14 for the CG, *p* < 0.05, paired t-test, see also Table 1). Re-evaluating KUT scores after the last session revealed no significant changes so that in the following sections we will refer to values of the first assessment on day one.

**Table 1:** **Grand average SMR power (in µV²) of the respective subgroups for electrode Cz across ten training sessions and while watching the feedback bars (baseline), as well as ratings of control beliefs on day one**.

		**EG**			**CG**		
		**all**	**low KUT**	**high KUT**	**all**	**low KUT**	**high KUT**
KUT	mean	32.7	29.0	36.4	33.0	29.4	36.6
	SEM	1.4	1.3	0.8	1.5	1.7	0.2
SMR training	mean	2.06	2.66	1.46	1.86	2.23	1.50
	SEM	0.26	0.35	0.09	0.33	0.62	0.23
SMR baseline	mean	1.97	2.46	1.48	1.94	2.39	1.48
	SEM	0.22	0.31	0.05	0.38	0.70	0.22

*EG, experimental group; CG control group; KUT, control beliefs while dealing with technology; SMR, sensorimotor rhythm; SEM, standard error of the mean; note that subgroups “low” and “high” of n = 5 participants were obtained using median split on KUT scores of “all” n = 10 participants.*.

**Figure 1:**
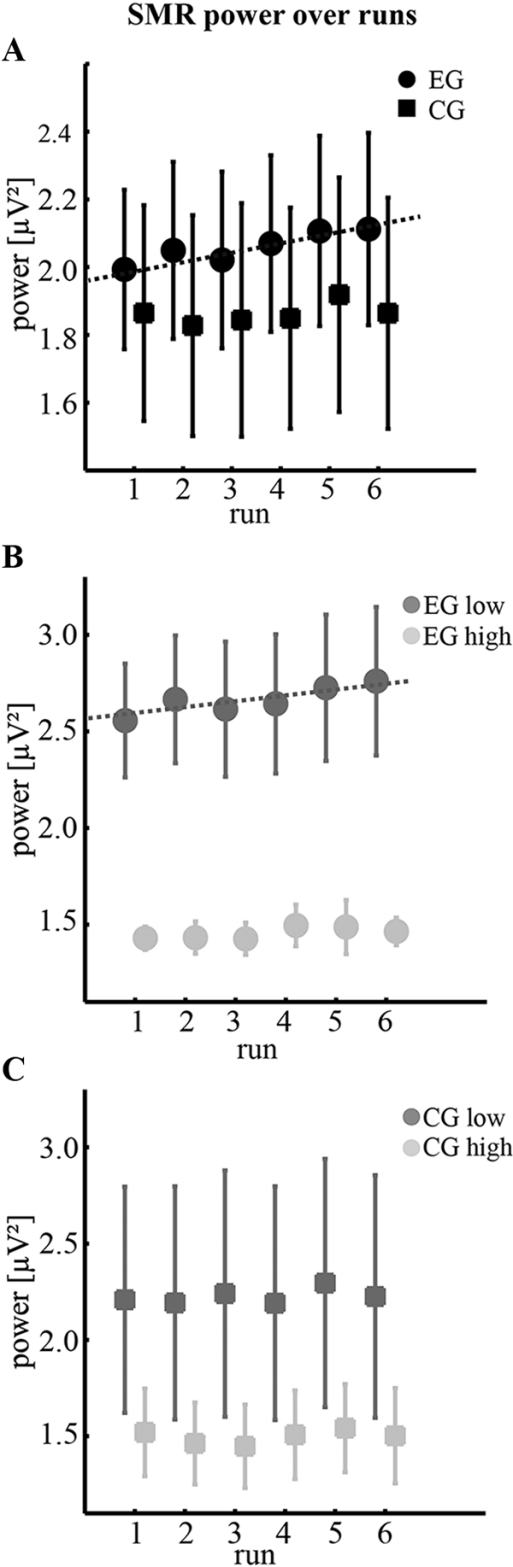
**Changes of SMR power during training**. **(A)** Mean absolute SMR power (12–15 Hz) across sessions during six runs of neurofeedback training for the experimental group using real feedback (EG, *n* = 10 participants) and the control group using sham feedback (CG, *n* = 10 participants). Dotted line indicates a significant slope of 0.023 µV^2^ per run. **(B,C)** Comparison of subgroups (*n* = 5 participants) obtained by median-split according to the individual control beliefs of low and high KUT scores. Dotted line indicates a significant slope of 0.035 µV^2^ per run. Note that all error bars represent the standard error of the mean (SEM).

Our main goal was to explore whether the observed differences in individual control beliefs were reflected in differential changes of SMR. Analysis of mean absolute SMR power did not reveal any significant effects over sessions. However, SMR power changed within session as indicated by the significant main effect of run (*F*(1, 18) = 4.51, *p* < 0.05, *η*^2^ = 0.20, *Obs_pow_* = 0.52). To further characterize this trend, we applied a linear fit for each group separately (Figure 1). While for the CG no trends were found (slope = 0.008, *p* = 0.35, *R*^2^ = 0.22, *n* = 10 participants), SMR of the EG consistently increased across runs (slope = 0.023, *p* < 0.01, *R*^2^ = 0.86, *n* = 10 participants). As evident in Figure 1B this effect was dominated by participants with low KUT scores (slope = 0.035, *p* < 0.05, *R*^2^ = 0.78, *n* = 5 participants) who also showed significantly higher SMR values when compared to participants of the EG with high KUT scores (*t*(8) = 3.37, *p* < 0.01, paired t-test). In contrast, this difference of SMR power between subgroups did not reach statistical significance for the CG (*t*(8) = 1.11, *p* = 0.30 n.s., paired t-test).

### Changes of SMR power in baseline

**Figure 2:**
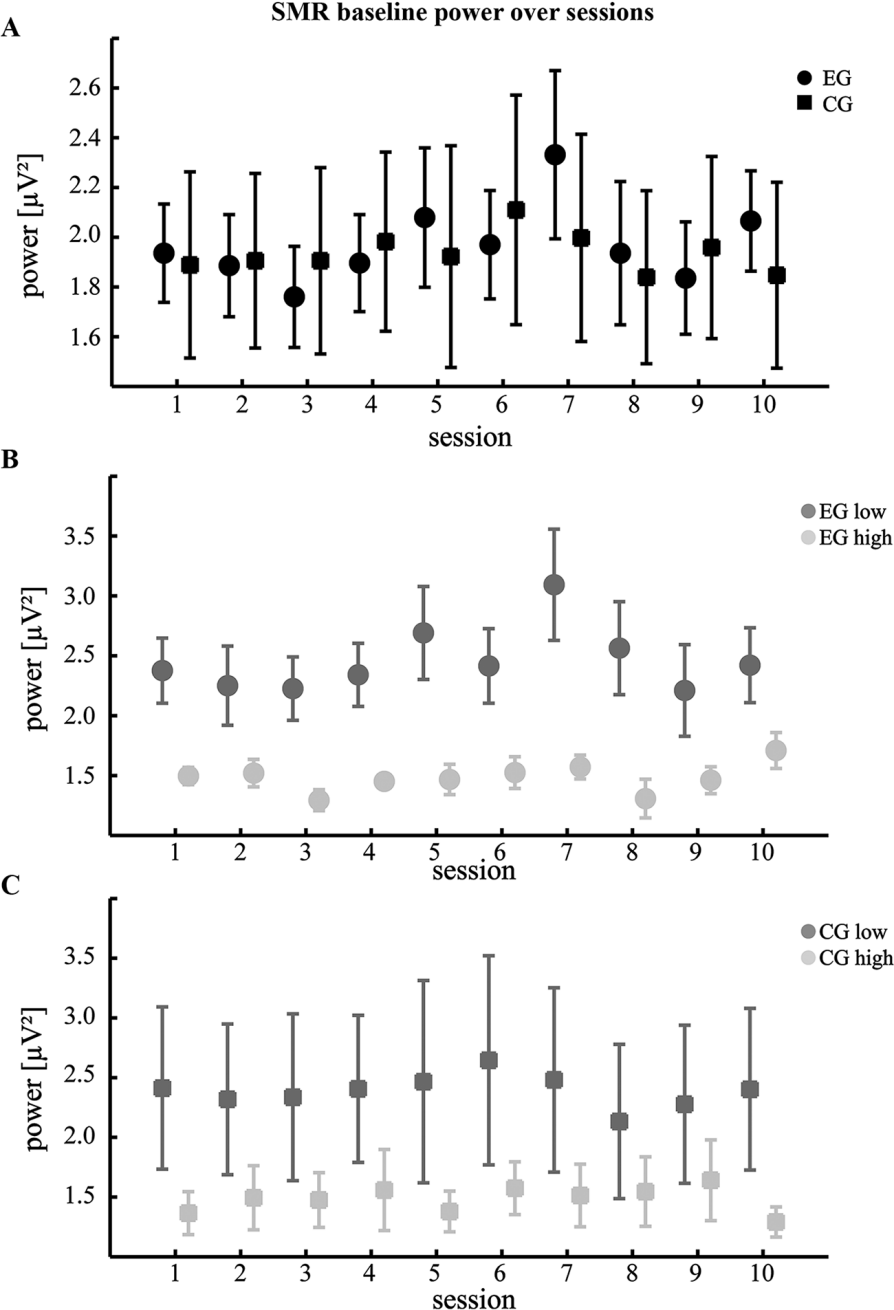
**Changes of SMR power during baseline**. **(A)** Mean absolute SMR power across 10 training sessions during the baseline condition. Participants of the EG were watching a visual feedback of their own brain activations without trying to gain control, while participants of the CG were watching a pre-recorded video **(B,C)** comparison according to the individual control beliefs (same conventions as in Figure 1).

To check for possible lasting effects of NF training we additionally compared baseline SMR power for the different groups. This analysis failed to show modulations across sessions. However, similar to the power changes over runs, we found that participants with lower KUT generally had increased SMR power compared to those with high KUT (Figure 2B and Table 1). Again, this difference was more pronounced for the EG (*t*(8) = 3.09, *p* < 0.05, paired t-test) when compared to the CG (*t*(8) = 1.24, *p* = 0.25 n.s., paired t-test).

### Overall correlation of KUT and SMR power

**Figure 3:**
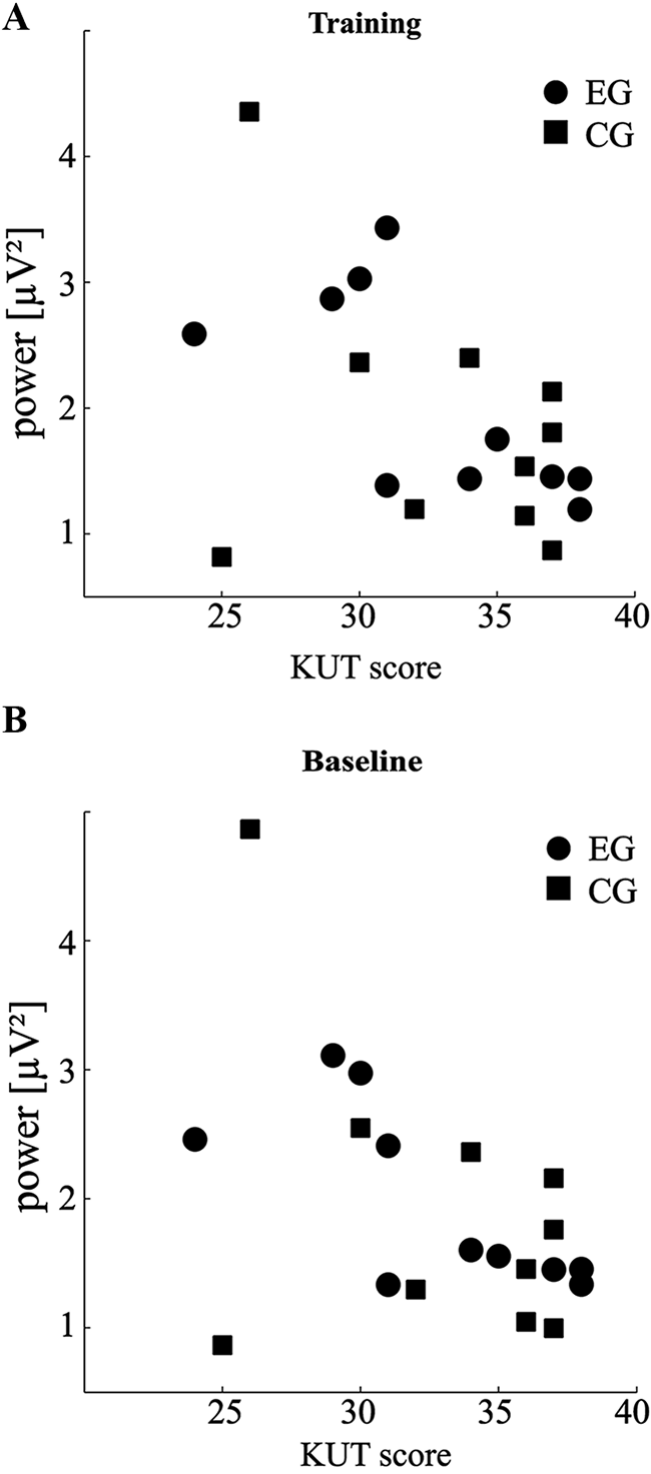
**SMR power correlates with control belief**. **(A)** Scatter plot of individual KUT scores against overall SMR power during feedback training (total *n* = 60 runs per participant). **(B)** Same as in **(A)** for baseline runs (total *n* = 10 runs per participant). For details of the relationships please see subsection “Overall Correlation of KUT and SMR Power” in Results.

The results described hitherto all indicate a trend for differences in SMR power between participants of low and high KUT scores. As a direct measure of this relationship we calculated Pearson’s linear correlation for both groups. This revealed a significant negative correlation between KUT and overall SMR power during training of *r* = −0.69 for the EG (*p* < 0.05). A strong trend for negative correlation of the same variables in the CG (*r* = −0.36, *p* = 0.31) was observed, only corrupted by one participant (Figure 3A).

As the within group differences in absolute SMR power were also evident in baseline, we additionally evaluated correlation coefficients between KUT and average SMR of these runs (Figure 3B). The overall picture was similar to training runs in that participants of the EG showed a negative correlation of *r* = −0.73 (*p* < 0.05) and participants of the CG displayed a similar trend (*r* = −0.42, *p* = 0.23).

## Discussion

As reliable predictors of NF performance still remain elusive, the goal of the current study was to assess whether control beliefs of users correlate with brain activity over several training sessions. Our main results show that voluntary up-regulation of absolute SMR power is more successful in those participants who report a lower subjective level of control while dealing with technology.

This novel finding is supported by several lines of evidence. Firstly, the overall power of SMR in the 12–15 Hz range across ten training sessions negatively correlated with KUT scores for the EG. In other words, participants with lower ratings of control belief were more successful in our training paradigm. According to Krampen (1981) control belief is defined as the individuals’ expectancy for a contingent result of an action. The KUT questionnaire quantifies a specific aspect of control, namely how comfortable and confident users feel when interacting with technology (Beier, 2004; Burde and Blankertz, 2006). How this psychological trait may be used to characterize NF performance has remained unexplored so far. Our findings of a negative correlation between KUT and SMR power indicate a higher relaxation in people who subjectively do not expect a major influence on technology. This state of relaxation in turn is known to promote increased SMR amplitudes (Pfurtscheller, 1992; Gruzelier et al., 2006, 2010; Pfurtscheller et al., 2006; Serruya and Kahana, 2008). At the same time our results imply that users with strong control beliefs may try harder to master the feedback paradigm and thus activate resources interfering with the SMR synchronization. This idea of “processing interference” has been proposed in healthy participants and seizure patients (Sterman, 1996, 2000).

In the literature there is only one study that assessed the link between control belief and modulation of brain signals: Burde and Blankertz (2006) reported a positive correlation between KUT and BCI performance. However, their task under investigation and the methods to reveal changes of brain activity clearly differ from our approach. These authors conducted a single session and relied on highly participant-optimized spectro-spatial features for providing feedback. Also they did not directly use the power of SMR for correlation analyses but rather assessed the number of runs in which participants successfully moved a cursor from the center to the target edge of a computer screen. Most importantly, this BCI control implied a motor imagery task where the classified pattern is that of a desynchronization of the SMR. In this light, participants with higher level of control belief could be more successful simply because they will likely try to actively control the feedback and thus down-regulate their SMR stronger during motor imagery. This decrease of activity is also depending on the level of SMR during rest (Blankertz et al., 2010a). In contrast, our NF paradigm directly trained the increase of absolute SMR power. In this task our findings suggest that stronger control beliefs can hinder participants’ relaxation and thus an effective up-regulation of SMR power during training. The ability of an individual user to succeed in both up- and down-regulation of SMR may therefore differ based on the different physiological processes and may furthermore depend on the task and method used to evaluate these modulations.

A second aspect of our training in the EG was an increase of SMR power over runs within sessions. Because we applied an online visual feedback, an overall higher level of relaxation may thus have promoted a self-rewarding positive loop. This within session increase is therefore believed to reflect successful training (Vernon et al., 2003; Gruzelier et al., 2006). The fact that absolute SMR power increased only moderately may partly originate from our experimental design: in line with suggestions of other studies (Kubler and Birbaumer, 2008; Nijboer et al., 2010) we adapted the difficulty of our task from run to run in the EG. While this procedure was believed to maintain a high level of motivation and interest in the task, past results also mentioned the risk of incompetence fear (Nijboer et al., 2008). In particular, these authors suggested that when performance in visual SMR feedback is initially high, incompetence fear may hamper further learning. Indeed, we do have evidence for a correlate of negative emotions in insular brain regions during our paradigm (Ninaus et al., submitted to the current special issue). How precisely emotions, task complexity and reward expectancy interact with performance thus needs to be explored in further studies. Interestingly, median split of participants of the EG revealed that those users with low KUT had distinctly higher SMR amplitudes over all runs and showed a significantly increasing trend. In our view, this corroborates the interpretation that control belief is directly linked to the success of SMR feedback training.

It has to be noted however that we did not find any significant modulations or interactions across training sessions. Yet, this does not conflict with past findings as those studies reporting inter-session changes either used ratios of the power within two or more frequency bands or relied on relative power changes (Ros et al., 2009; Gruzelier et al., 2010; de Zambotti et al., 2012). In contrast, our measure of absolute SMR power represents a direct index of brain activity. A distinct increase of this index over sessions actually would not have been expected. de Zambotti et al. (2012) recently reported an increase of the SMR-*θ* ratio across weeks of NF training. However, this ratio was calculated with respect to a baseline and authors mentioned a decrease of SMR-*θ* in this baseline. As a consequence, the observed training effect in the study of de Zambotti et al. (2012) may not have been solely caused by brain processes in the active period. Our results during baseline did not show a change of SMR power ruling out the possibility of a pseudo training effect. Yet, we again observed a markedly higher SMR for participants of low KUT scores underlining the general validity of this relationship. An important difference to previous work is that during baseline in our approach participants watched feedback bars coupled to their actual brain activity without trying to control the feedback bars. This might explain why overall baseline SMR power was not different to training runs. The role of baseline recordings in assessing changes of power over sessions may thus need further research.

To check for the task-specificity of the observed effects, we included the CG receiving a video of sham feedback. Although the overall SMR power was not significantly different to the EG and since a strong trend for a negative correlation between KUT and SMR was evident, some important points should be considered. First, participants of the CG tended to show lower power values and a higher variance across participants was observed (Figures 1,2). Second, there was no clear increase of power over runs (Figure 1C). And third, the within-group difference between participants of low and high KUT scores was less pronounced than in the EG. Altogether, we thus conclude that in contrast to other studies our CG experienced a residual, although less effective, training as well. This is supported by the fact that no member of this group identified the sham feedback. Instead, the randomized replay of moving bars was accepted as real feedback so that a similar pattern of the relationship between KUT and SMR power emerged. The greater amount of variability and the non-significant dissociation of SMR power between KUT subgroups of the CG suggest, however, that only contingent feedback training can produce clear task-specific effects.

Our findings support the existing NF literature that has suggested a state of relaxed but focused mind for successful performance. For example, a recent study showed a strong inhibition of SMR in initial sessions which was attributed to increased arousal of participants who most likely needed to get used to the experimental setup (de Zambotti et al., 2012). Hammer and colleagues (Hammer et al., 2012) also reported that, besides fine motor skills, the ability to concentrate on the task explained a significant proportion of 19% of the variations seen in BCI performance. At the same time all other psychological parameters, like verbal and non-verbal learning abilities, empathy or mood, did not predict performance in this study. Similarly, motivational factors seem to be only weak predictors of performance and need to be considered at a single subject level (Nijboer et al., 2008, 2010; Kleih et al., 2010). Still, the finding that initial performance during voluntary regulation of brain activity has predictive value (Neumann and Birbaumer, 2003; Kübler et al., 2004) may suggest that personal traits can impact the ability to successfully use feedback paradigms. One important factor in NF and BCI is clearly the individual control beliefs of participants because the tasks per se imply the interaction with technology. The only study in this context we are aware of has already demonstrated a strong correlation between control belief and BCI performance. We clearly extend this knowledge as we identified control beliefs while dealing with technology as a strong predictor of performance in several training sessions. Whether our findings generalize to other frequency-bands and experimental setups needs further validation. In studies that focus on brain activity associated with relaxation, e.g., in treatment of attention deficit hyperactivity disorder, one would expect similar predictive effects. In general, we strongly suggest that concepts of control belief, self-perception and awareness should be considered in more detail during BCI and NF operation.

## Conclusion

In summary, we demonstrate that control beliefs negatively correlated with the ability to increase SMR during 10 NF sessions. An important implication for future training studies therefore is that participants may not focus on gaining control over the feedback but instead should try to relax themselves. In the light of our results, assessment of individual control beliefs can be used as a predictor of future performance and may thus help to avoid lengthy training.

## Conflict of interest statement

The authors declare that the research was conducted in the absence of any commercial or financial relationships that could be construed as a potential conflict of interest.
